# Integrative Genomic and Transcriptomic Analysis Identifies BAX as a Prognostic Marker of Disease Progression in Prostate Cancer

**DOI:** 10.3390/genes17070804

**Published:** 2026-07-15

**Authors:** You-Cheng Shih, Chi-Fen Chang, Chao-Yuan Huang, Chia-Cheng Yu, Victor C. Lin, Te-Ling Lu, Shu-Pin Huang, Bo-Ying Bao

**Affiliations:** 1 Department of Pharmacy, China Medical University, Taichung 406, Taiwan; u112003455@cmu.edu.tw (Y.-C.S.); lutl@mail.cmu.edu.tw (T.-L.L.); 2 Department of Anatomy, School of Medicine, China Medical University, Taichung 406, Taiwan; cfchang@mail.cmu.edu.tw; 3 Department of Urology, National Taiwan University Hospital, College of Medicine, National Taiwan University, Taipei 100, Taiwan; cyh0909@ntu.edu.tw; 4 Division of Urology, Department of Surgery, Kaohsiung Veterans General Hospital, Kaohsiung 813, Taiwan; ccyu@vghks.gov.tw; 5 Department of Urology, School of Medicine, National Yang Ming Chiao Tung University, Taipei 112, Taiwan; 6 Department of Pharmacy, College of Pharmacy and Health Care, Tajen University, Pingtung 907, Taiwan; 7 Department of Urology, E-Da Hospital, Kaohsiung 824, Taiwan; ed102161@edah.org.tw; 8 School of Medicine for International Students, I-Shou University, Kaohsiung 840, Taiwan; 9 Graduate Institute of Clinical Medicine, College of Medicine, Kaohsiung Medical University, Kaohsiung 807, Taiwan; 10 Department of Urology, Kaohsiung Medical University Hospital, Kaohsiung 807, Taiwan; 11 Institute of Medical Science and Technology, College of Medicine, National Sun Yat-sen University, Kaohsiung 804, Taiwan

**Keywords:** prostate cancer, androgen deprivation therapy, endoplasmic reticulum stress, BAX, biomarker

## Abstract

**Background/Objectives**: Prostate cancer is one of the most common malignancies among men worldwide, and clinical outcomes following androgen deprivation therapy (ADT) vary considerably. Given that endoplasmic reticulum (ER) stress and the unfolded protein response mediate processes such as apoptosis, tumor adaptation, and disease progression, we aimed to investigate whether genetic variants in ER stress-related genes are associated with survival outcomes in patients with prostate cancer receiving ADT. **Methods**: This study enrolled 630 patients with prostate cancer who underwent ADT across three medical centers in Taiwan. A genetic association analysis of 89 haplotype-tagged single-nucleotide polymorphisms (SNPs) across 12 ER stress-related genes was performed. The primary clinical endpoint was overall survival (OS). Kaplan–Meier survival analysis and Cox proportional hazards models were used to evaluate prognostic associations. Furthermore, publicly available databases were integrated to analyze gene expression, clinical relevance, gene set enrichment, and tumor immune infiltration to elucidate the underlying biological mechanisms. **Results**: Among the analyzed SNPs, *BAX* rs182509214 showed the strongest association with OS. The minor G allele of *BAX* rs182509214 was significantly associated with poorer OS. Prostate tumor tissues exhibited markedly elevated *BAX* expression compared with normal prostate tissues, and this elevated expression was associated with worse survival outcomes. Multiple public gene expression datasets confirmed the overexpression of *BAX* in prostate cancer. Functional analyses revealed that genes associated with *BAX* expression were predominantly enriched in ribosomal, oxidative phosphorylation, and proteasomal pathways. Furthermore, the *BAX* copy number variation was significantly associated with the infiltration levels of multiple immune cell types, and *BAX* expression was negatively correlated with CD8^+^ T-cell infiltration, implying a potential marker role for the tumor immune microenvironment. **Conclusions**: The ER stress-related genetic variant, *BAX* rs182509214, may influence survival outcomes in patients with prostate cancer receiving ADT. *BAX* alterations are associated with disease progression and linked to mitochondria-related metabolic pathways and the tumor immune microenvironment. These results highlight *BAX* as a potential prognostic biomarker for prostate cancer treated with ADT; however, further validation in larger cohorts and functional studies is warranted.

## 1. Introduction

Prostate cancer represents a substantial global health burden, with its incidence increasing notably in middle-aged and older men. Approximately 1.5 million individuals were newly diagnosed in 2022, and approximately 400,000 died from the disease, underscoring its status as a leading global malignancy [1]. As prostate tumor growth depends heavily on androgen signaling, androgen deprivation therapy (ADT) remains the cornerstone of treatment for advanced and metastatic diseases, functioning by lowering systemic testosterone levels and limiting androgen receptor (AR) activation [2,3]. Clinically, ADT is achieved using several strategies, including surgical castration, luteinizing hormone-releasing hormone (LHRH) agonists, LHRH antagonists, and AR antagonists [3,4]. Despite these established approaches, patient outcomes vary widely. Recurrence risk differs markedly among individuals, and conventional predictors, such as prostate-specific antigen (PSA) levels, tumor stage, and Gleason scores, do not fully capture this heterogeneity [5,6]. Accumulating evidence indicates that genetic alterations, including mutations in genes critical to prostate cancer pathogenesis (e.g., *TP53*, *PTEN*, and DNA damage–repair genes), can substantially increase the risk of recurrence [7]. Nevertheless, clinical management remains limited owing to suboptimal early risk stratification and the frequent emergence of therapeutic resistance. This highlights the need to integrate genetic determinants with clinical factors to refine prognostic models and facilitate personalized care.

At the molecular level, the endoplasmic reticulum (ER) orchestrates protein folding and post-translational processing. When misfolded proteins accumulate beyond the capacity of the ER, cells initiate the unfolded protein response (UPR), which may ultimately trigger apoptosis if homeostasis cannot be restored [8]. Among the major UPR branches, the inositol-requiring enzyme 1α (IRE1α) pathway functions as a key stress sensor and signaling hub [9]. Under basal conditions, the chaperone heat shock protein family A member 5 (HSPA5) maintains IRE1α in an inactive state; during ER stress, HSPA5 dissociates, allowing IRE1α to undergo oligomerization, activation, and autophosphorylation. Activated IRE1α subsequently recruits TNF receptor-associated factor 2 (TRAF2), which activates mitogen-activated protein kinase kinase kinase 5 (MAP3K5) and the downstream mitogen-activated protein kinase kinase 4 (MAP2K4) and 7 (MAP2K7), culminating in activation of mitogen-activated protein kinase 8 (MAPK8), a central mediator of ER stress-induced apoptosis [10,11,12,13,14]. Concurrently, activated IRE1α catalyzes the unconventional splicing of X-box binding protein 1 (XBP1) mRNA to generate the active transcription factor XBP1s, which promotes adaptive responses that enhance protein folding, secretion, and ER-associated degradation [11]. When ER stress is prolonged or excessive, DNA damage-inducible transcript 3 (DDIT3) suppresses anti-apoptotic signaling while inducing the expression and activation of pro-apoptotic BCL-2 family members, including BCL2 like 11 (BCL2L11), BCL2 associated X (BAX), and BCL2 antagonist/killer 1 (BAK1), thereby promoting mitochondrial outer membrane permeabilization and apoptosis [15]. In parallel, DDIT3 also transcriptionally upregulates TNF receptor superfamily member 10B (TNFRSF10B), linking prolonged ER stress to death receptor-mediated apoptosis [16]. In addition to these canonical ER stress components, advillin (AVIL), an actin-binding protein involved in cytoskeletal remodeling and cell motility, has recently been implicated in cancer progression and metastatic behavior, suggesting that it may contribute to cellular adaptation under stress conditions [17]. Genetic variations affecting these ER stress- and apoptosis-related genes may alter gene regulation or protein function, thereby influencing cancer susceptibility, disease progression, and therapeutic responses. Consistent with this concept, single-nucleotide polymorphisms (SNPs) in ER stress-related genes have been associated with cancer susceptibility and clinical outcomes in several malignancies [18].

Although ADT is often initially effective, many patients eventually develop castration-resistant prostate cancer (CRPC), a lethal state characterized by androgen-independent tumor growth. Following CRPC diagnosis, survival is frequently limited to 20–24 months [19]. Given the pronounced variability in ADT efficacy, pharmacogenomic approaches have increasingly focused on identifying genetic markers that modulate tumor progression and drug response [20,21,22]. In this context, we hypothesized that inherited variants within the ER stress and UPR pathways influence prostate cancer progression and therapeutic outcomes in the setting of ADT. Accordingly, this study evaluated SNPs in ER stress-related genes to examine their association with overall survival (OS) in patients with prostate cancer receiving ADT, with the aim of identifying SNP-based predictors to improve risk stratification and inform biomarker development.

## 2. Materials and Methods

### 2.1. Study Population and Clinical Data

We enrolled 630 patients with confirmed prostate cancer who received ADT at three medical centers in Taiwan: National Taiwan University Hospital, Kaohsiung Medical University Hospital, and Kaohsiung Veterans General Hospital [23,24]. The Institutional Review Board of Kaohsiung Medical University Hospital approved the study protocol (KMUHIRB-2013132), and the study was conducted in accordance with the Declaration of Helsinki and Good Clinical Practice guidelines. Written informed consent was obtained from all participants before enrollment. Clinical and pathological data were retrospectively retrieved from institutional medical records. The primary outcome was OS, defined as the time from ADT initiation to death from any disease. During the median follow-up period of 165.8 months, 414 deaths were documented (Table S1). Key clinicopathological variables, including age, PSA levels at ADT initiation, clinical stage, Gleason scores, PSA nadir, and time to PSA nadir, were significantly associated with OS (*p* < 0.05).

### 2.2. SNP Selection and Genotyping

Haplotype-tagged SNPs were selected from 12 ER stress-related genes using Haploview version 4.2 and the Tagger algorithm [25]. The candidate genes comprised *AVIL*, *BAK1*, *BAX*, *BCL2L11*, *DDIT3*, *MAP2K4*, *MAP2K7*, *MAP3K5*, *MAPK8*, *TNFRSF10B*, *TRAF2*, and *XBP1*. Variants were selected based on Han Chinese genotype data from the 1000 Genomes Project (Beijing Han Chinese and Southern Han Chinese cohorts), using a minor allele frequency (MAF) threshold of > 0.05 and a linkage disequilibrium criterion of *r*^2^ > 0.8. Genomic DNA was extracted from whole-blood samples using the QIAamp DNA Blood Kit (Qiagen, Germantown, MD, USA). Genotyping was performed at the National Center for Genome Medicine of Taiwan using the Affymetrix Axiom Genotyping Array system (Thermo Fisher Scientific, Waltham, MA, USA) [26]. SNPs were excluded if they exhibited an MAF < 0.03, a genotyping call rate < 0.94, or a significant deviation from Hardy–Weinberg equilibrium (*p* < 0.0001). Ultimately, 89 SNPs were retained for downstream analysis.

### 2.3. Bioinformatic and Transcriptomic Analyses

To determine *BAX* expression and its clinical relevance in prostate cancer, we analyzed publicly available datasets, including PCaDB [27], the Gene Expression Database of Normal and Tumor Tissues 2 (GENT2) [28], and The Cancer Genome Atlas Prostate Adenocarcinoma (TCGA-PRAD) cohort. The LinkedOmics database was used to identify genes correlated with *BAX* expression and to explore potential biological mechanisms [29]. Gene Set Enrichment Analysis (GSEA) was performed to assess the enrichment of Gene Ontology (GO) terms and Kyoto Encyclopedia of Genes and Genomes (KEGG) pathways. Genes were ranked according to their Pearson’s correlation coefficients relative to *BAX* expression. Furthermore, the Tumor Immune Estimation Resource 2.0 (TIMER2.0) was employed to evaluate *BAX* somatic copy number alterations (SCNAs), which were categorized as deep deletion (−2), arm-level deletion (−1), diploid (0), arm-level gain (1), or high amplification (≥2). Additionally, TIMER2.0 was used to quantify immune infiltration and to examine the correlation between *BAX* expression and the relative abundance of immune cell populations [30]. The data analyzed in this study were retrieved from the respective databases as available on 18 Octorber 2025.

### 2.4. Statistical Analysis

All statistical analyses were performed using SPSS version 19.0.0 (IBM, Armonk, NY, USA). Statistical significance was established at a two-sided *p*-value < 0.05. Survival curves were generated using the Kaplan–Meier method, and intergroup differences were evaluated using the log-rank test. Cox proportional hazards regression models were used to assess associations between clinicopathological variables and patient prognosis, and results were reported as hazard ratios (HRs) with 95% confidence intervals (CIs). Spearman and Pearson’s correlation coefficients were used to assess relationships between *BAX* expression and specific tumor characteristics. Finally, a pooled analysis comparing *BAX* expression in prostate cancer tissues with that in normal tissues was performed using the Review Manager version 5.4.1 (Cochrane, London, UK). These findings were summarized as standardized mean differences (SMDs) with corresponding 95% CIs.

## 3. Results

### 3.1. ER Stress-Related SNPs and Survival Analysis

**Figure 1:**
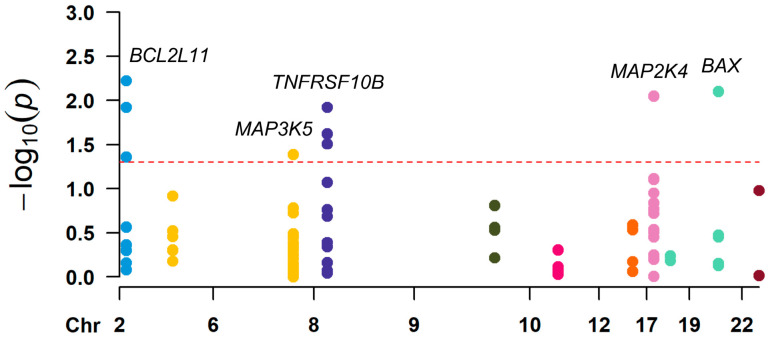
Association of endoplasmic reticulum stress-related single-nucleotide polymorphisms (SNPs) with overall survival. Manhattan plot of the associations between 89 SNPs across 12 genes and overall survival in patients with prostate cancer undergoing androgen deprivation therapy. Chromosomal positions are plotted on the x-axis against −log10(*p*) values on the y-axis. Dots of different colors represent SNPs located in different chromosomal regions to facilitate visualization of adjacent loci. The horizontal red dashed line indicates nominal significance (*p* = 0.05), with significant genes labeled.

**Figure 2:**
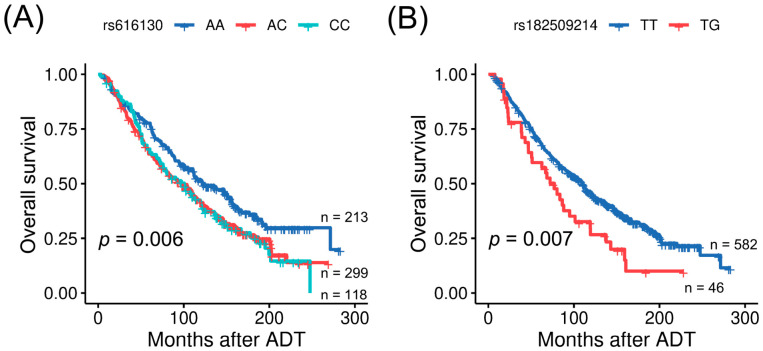
Kaplan–Meier overall survival curves stratified by *BCL2L11* and *BAX* genotypes. Survival probabilities for patients with prostate cancer receiving androgen deprivation therapy (ADT) are shown according to the genotypes of (**A**) *BCL2L11* rs616130 and (**B**) *BAX* rs182509214.

To examine the relationship between the ER stress pathway and prostate cancer progression, we analyzed 12 implicated genes to determine their impact on OS following ADT. Among the evaluated SNPs, nine were significantly associated with OS (*p* < 0.05; Figure 1), including rs3761704, rs616130, and rs10189015 in *BCL2L11*; rs147842470 in *MAP3K5*; rs7834266, rs56070946, and rs1001792 in *TNFRSF10B*; rs28922868 in *MAP2K4*; and rs182509214 in *BAX*. The SNPs *BCL2L11* rs616130 and *BAX* rs182509214 demonstrated the most significant associations with OS. Specifically, the minor allele C of *BCL2L11* rs616130 was associated with an increased risk of all-cause mortality compared with the major allele A (HR = 1.20, 95% CI = 1.05–1.37, *p* = 0.006, false discovery rate [FDR] = 0.214; Table 1 and Figure 2A). Similarly, the minor allele G of *BAX* rs182509214 was associated with a heightened risk of mortality (HR = 1.60, 95% CI = 1.13–2.27, *p* = 0.008, FDR = 0.214; Table 1 and Figure 2B). However, in multivariate analysis adjusted for clinical variables, including age, PSA level at ADT initiation, clinical stage, Gleason score, PSA nadir, and time to PSA nadir, only *BAX* rs182509214 remained an independent predictor of OS (HR = 1.68, 95% CI = 1.18–2.40, *p* = 0.004).

### 3.2. Elevated BAX Expression in Prostate Tumor Cells

**Figure 3:**
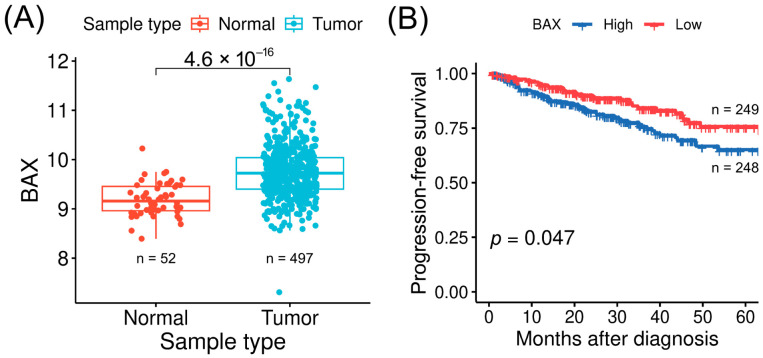
Expression analysis of *BAX* in prostate cancer. (**A**) *BAX* expression levels in prostate tumor tissues compared with corresponding normal prostate tissues, based on data from The Cancer Genome Atlas Prostate Adenocarcinoma cohort. (**B**) Association between *BAX* expression levels and survival outcomes in patients with prostate cancer.

To explore the clinical implications of *BAX*, we analyzed its expression levels in prostate tumors and normal prostate tissues using the TCGA-PRAD dataset. *BAX* expression was significantly elevated in prostate tumors compared with normal tissues (*p* = 4.6 × 10^−16^; Figure 3A). Furthermore, high *BAX* expression was associated with poorer survival outcomes in patients with prostate cancer (*p* = 0.047; Figure 3B).

**Figure 4:**
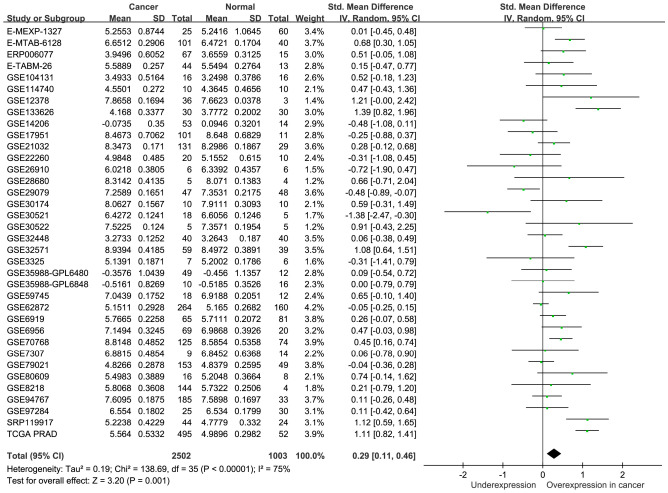
Forest plot illustrating the differential expression of *BAX* between prostate tumor tissues and normal prostate tissues across multiple independent transcriptomic datasets.

To validate the clinical relevance of *BAX*, we conducted a pooled analysis of *BAX* expression across 36 publicly available transcriptomic datasets encompassing 2502 prostate tumor samples and 1003 normal prostate tissues. This pooled analysis confirmed significantly higher *BAX* expression in prostate cancer tissues compared with normal prostate tissues (SMD = 0.29, 95% CI = 0.11–0.46, *p* = 0.001; Figure 4).

### 3.3. Functional Enrichment of BAX-Associated Genes in Prostate Cancer

**Figure 5:**
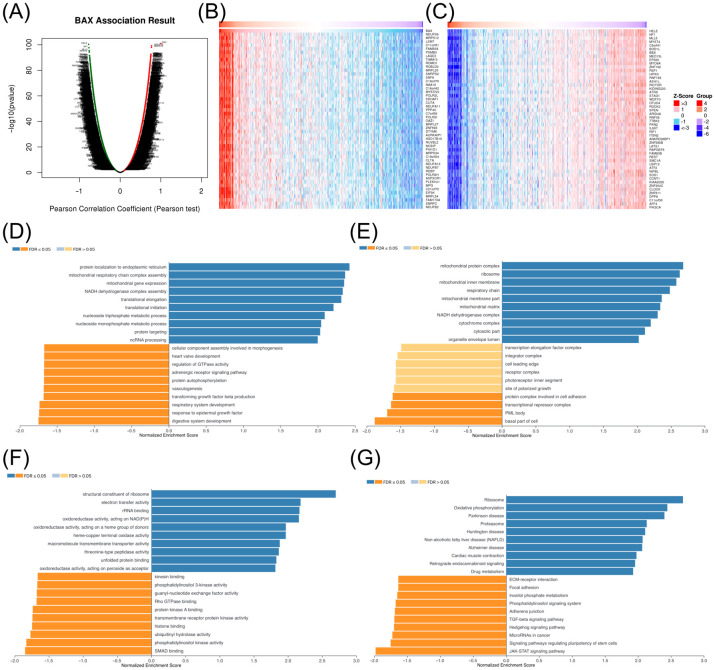
Functional enrichment and co-expression analysis of *BAX*-associated genes using LinkedOmics. (**A**) Volcano plot of genes correlated with *BAX* expression, determined via Pearson correlation analysis. Heatmaps displaying the top 50 (**B**) positively and (**C**) negatively correlated genes with *BAX* in prostate cancer. (**D**–**F**) Gene Set Enrichment Analysis of *BAX*-associated genes categorized by (**D**) biological processes, (**E**) cellular components, and (**F**) molecular functions. (**G**) KEGG pathway enrichment analysis of *BAX*-associated genes.

To investigate the biological significance of *BAX* in prostate cancer, we used the LinkedOmics database to analyze gene co-expression correlations within the TCGA-PRAD cohort. We identified 5654 positively correlated genes and 7029 negatively correlated genes associated with *BAX* expression (Figure 5A). The top 50 positively and negatively correlated genes were visualized using heatmaps (Figure 5B,C).

GO analysis indicated that *BAX*-associated biological processes included protein localization to the ER, mitochondrial respiratory chain complex assembly, and mitochondrial gene expression (Figure 5D). The enriched cellular components included the mitochondrial protein complex, ribosomes, mitochondrial inner membrane, and respiratory chain (Figure 5E). Significant molecular functions included structural constituents of the ribosome, electron transfer activity, and rRNA binding (Figure 5F). Furthermore, KEGG pathway enrichment analysis revealed that *BAX* was strongly associated with ribosomal, oxidative phosphorylation, and proteasomal pathways (Figure 5G). Collectively, these findings suggest that *BAX* is involved in diverse mitochondrial- and ribosome-related biological processes that may facilitate cancer cell survival.

### 3.4. Analysis of BAX-Associated Immune Cell Infiltration in the Tumor Microenvironment

**Figure 6:**
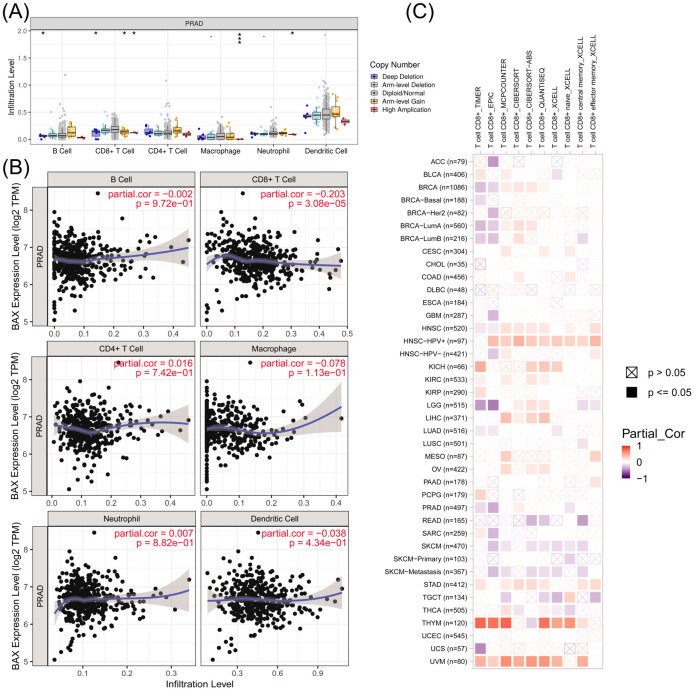
Association between *BAX* alterations and immune cell infiltration levels in the prostate cancer tumor microenvironment. Analyses were performed using the Tumor Immune Estimation Resource database. (**A**) Impact of *BAX* somatic copy number alterations on the infiltration levels of diverse immune cell populations in prostate tumors. Statistical significance: *** *p* < 0.001; * *p* < 0.05. (**B**) Correlation between *BAX* expression and CD8^+^ T-cell infiltration levels in prostate cancer. (**C**) Association between *BAX* expression and CD8^+^ T-cell infiltration levels across various cancer types.

To further investigate the relationship between *BAX* and the tumor immune microenvironment (TIME) in prostate cancer, we used the TIMER database to evaluate the immunological impact of *BAX* expression. Analysis using the SCNA module revealed that *BAX* copy number variations were significantly associated with the infiltration levels of multiple immune cell populations, including B cells, CD8^+^ T cells, macrophages, and neutrophils (Figure 6A). Further analyses demonstrated that *BAX* expression was negatively correlated with CD8^+^ T-cell infiltration in prostate tumors, with a partial correlation of −0.203 (*p* = 3.08 × 10^−5^; Figure 6B). This inverse association was also observed in several other malignancies, including breast invasive carcinoma, head and neck squamous cell carcinoma, low-grade glioma, and uterine serous carcinoma (Figure 6C). Collectively, these findings suggest that *BAX* genomic alterations and overexpression inversely correlate with cytotoxic immune cell infiltration, indicating an association with an immunosuppressive tumor microenvironment and disease progression.

## 4. Discussion

This study provides evidence that the ER stress-related gene *BAX* and its genetic variant rs182509214 are associated with the prognosis of patients with prostate cancer receiving ADT. Specifically, the minor G allele of rs182509214 was associated with markedly poorer OS, supporting the utility of germline variation as a prognostic biomarker in this clinical context. Furthermore, analyses of publicly available transcriptomic datasets demonstrated that *BAX* was substantially overexpressed in prostate tumor tissues compared with normal prostate tissues, consistent with previous reports implicating dysregulated *BAX* expression in tumor development and progression.

From a population genetics perspective, rs182509214 is a *BAX* variant with a measurable MAF of 0.02 in Asian populations. HaploReg annotations indicated that this locus overlaps with regions enriched for promoter- and enhancer-associated histone marks, as well as DNase I hypersensitive sites, suggesting that it resides within a transcriptionally active regulatory element. Furthermore, this locus exhibits signals for RNA polymerase II binding and is associated with alterations in multiple transcriptional regulatory motifs. Collectively, these in silico annotations suggest that rs182509214 lies within an active regulatory region and may modulate the transcriptional control of *BAX*. Nevertheless, because HaploReg integrates epigenomic signals across diverse tissues, prostate-specific regulatory activity and precise genotype-expression relationships must be validated using prostate-relevant quantitative trait loci resources and functional assays, such as luciferase reporter assays and allele-specific chromatin immunoprecipitation quantitative polymerase chain reaction.

Physiologically, *BAX* is a critical downstream effector of ER stress-mediated apoptosis. Severe ER stress, induced by the accumulation of unfolded or misfolded proteins, triggers the UPR via branches such as PERK–eIF2α–ATF4 and IRE1–JNK signaling. These pathways can activate pro-apoptotic transcriptional programs (e.g., DDIT3 and ATF4) that upregulate BCL-2 family members, including *BAX*, ultimately promoting mitochondrial outer membrane permeabilization (MOMP) and subsequent apoptosis [31]. Consistent with our transcriptomic analyses of public cohorts, higher *BAX* expression in prostate cancer was paradoxically associated with poorer survival outcomes [32,33]. This elevated *BAX* mRNA abundance may reflect a compensatory cellular stress response or transcriptional upregulation, rather than increased apoptosis. To investigate this, we examined the TCGA-PRAD proteomic dataset and observed a significant but modest positive correlation between *BAX* mRNA and protein expression (Spearman’s *r* = 0.282, *p* < 0.001). This indicates that while transcriptional upregulation partially translates into increased protein abundance, BAX is subject to substantial post-transcriptional and post-translational regulation. Mechanistically, BAX protein activity can be antagonized by TMBIM6 or attenuated through ubiquitin–proteasome-mediated degradation [34,35]. Furthermore, we examined the expression of the anti-apoptotic gene *BCL2L1* (BCL-xL) to understand potential compensatory mechanisms. Prostate tumors exhibited significantly higher *BCL2L1* expression than normal prostate tissues (*p* < 0.001), and tumors with high *BAX* expression showed concurrently elevated *BCL2L1* expression (*r* = 0.272, *p* < 0.001). Because BCL2L1 directly binds and neutralizes activated BAX, preventing MOMP and apoptosis, these findings suggest that increased *BAX* expression is accompanied by a compensatory increase in anti-apoptotic signaling. Consequently, elevated *BAX* expression does not necessarily translate into effective apoptotic execution, providing a plausible explanation for its association with adverse clinical outcomes despite its canonical pro-apoptotic function. Taken together, these post-transcriptional, post-translational, and counter-regulatory mechanisms suggest that elevated *BAX* transcript levels may serve as a surrogate marker for aggressive tumor biology and adverse OS, rather than reflecting an intact and effective apoptotic response. Supporting the broader clinical relevance of *BAX* genetic variations, G/A polymorphisms in the *BAX* promoter have been associated with altered BAX protein expression, malignant phenotypes, and reduced OS in esophageal cancer [36]. Furthermore, specific *BAX* variants in non-small cell lung cancer have been shown to predict inferior outcomes and shorter progression-free and OS in patients receiving platinum-based chemotherapy [37]. Taken together, these observations reinforce the hypothesis that *BAX* dysregulation and its associated genetic variants influence tumor progression and clinical trajectories across multiple malignancies, including prostate cancer [38,39].

Beyond tumor-intrinsic apoptosis, *BAX* has been increasingly implicated in shaping the immunoregulatory landscape of the TIME. Under specific conditions, particularly when caspase activity is compromised, *BAX*-mediated MOMP facilitates the release of mitochondrial DNA into the cytosol, thereby activating the cGAS–STING signaling pathway and inducing type I interferon (IFN) and inflammatory signaling. In the context of the TIME, chronic IFN signaling frequently drives immunosuppressive adaptations, including the upregulation of immune checkpoints such as PD-L1, resulting in an “inflamed-but-suppressed” state of adaptive immune resistance [40]. To determine whether this phenotype is present in our study context, we performed additional analyses using the TCGA-PRAD cohort. Our results showed that patients with high *BAX* expression exhibited elevated expression of interferon-stimulated genes, including ISG15 ubiquitin-like modifier (*r* = 0.334, *p* < 0.001) and interferon regulatory factor 7 (IRF7; *r* = 0.462, *p* < 0.001), consistent with activation of the IFN pathway. Concurrently, high *BAX* expression was associated with upregulation of immune checkpoint molecules, including cytotoxic T-lymphocyte-associated protein 4 (CTLA4; *r* = 0.096, *p* = 0.033) and lymphocyte-activation gene 3 (LAG3; *r* = 0.189, *p* < 0.001), suggesting a pronounced adaptive immunosuppressive response accompanying the initial inflammatory signal. By contrast, *CD8A* expression, used as a proxy for CD8+ T-cell abundance, was negatively correlated with *BAX* expression (*r* = −0.097, *p* = 0.030). This finding directly supports our initial observations from the TIMER database, which indicated reduced CD8+ T-cell infiltration. Consistent with this paradigm, pan-cancer analyses have indicated that elevated *BAX* expression correlates with both increased immune cell infiltration and the concurrent upregulation of immunosuppressive checkpoint networks, suggesting that cell death signatures often co-occur with an immunosuppressed TIME. For instance, in gliomas, a pyroptosis-related expression phenotype characterized by high *BAX* expression has been associated with immunosuppressive TIME features (e.g., accumulation of M2 macrophages, myeloid-derived suppressor cells, and regulatory T cells) and poorer survival outcomes [41]. Although specific evidence in prostate cancer remains limited, the interplay between *BAX*, TIME remodeling, and clinical outcomes warrants rigorous experimental validation.

GSEA identified a positive association between *BAX* expression and ribosome-related pathways, likely reflecting the coordinated upregulation of mitochondrial ribosomal components and energy metabolism pathways. Notably, mitochondrial ribosomal protein S12 (*MRPS12*) emerged as a representative hub gene within this signature (Pearson *r* = 0.770, *p* = 1.10 × 10^−98^). As a mitochondrial ribosomal protein, *MRPS12* is integral to mitochondrial protein translation and oxidative phosphorylation; thus, its upregulation may reflect the heightened metabolic demands characteristic of rapid tumor proliferation [42]. Previous studies have demonstrated that high *MRPS12* expression is associated with enrichment of pathways involved in dysregulated growth control, including the G2/M checkpoint, PI3K/AKT/mTOR signaling, and p53 pathways. Clinically, *MRPS12* overexpression is associated with adverse prognosis in ovarian and non-small cell lung cancers, highlighting its potential utility as a prognostic biomarker [43,44]. Collectively, these findings support the hypothesis that *BAX*-associated mitochondrial translation and metabolic programs may be associated with prostate cancer progression. However, the precise mechanistic role of *MRPS12* in prostate cancer, particularly under the selective pressure of ADT, remains to be elucidated and requires further investigation and experimental validation.

In addition to *MRPS12*, NADH:ubiquinone oxidoreductase subunit S6 (*NDUFS6*) and LSM7 homolog, U6 small nuclear RNA and mRNA degradation associated (*LSM7*) were identified as representative genes with high *BAX* expression enrichment patterns, suggesting that *BAX*-associated transcriptional programs encompass post-transcriptional RNA regulation, oxidative phosphorylation, and mitochondrial translation. NDUFS6, a subunit of the mitochondrial electron transport chain complex I, is essential for cellular respiration and electron transport. In melanoma, NDUFS6 interacts with CD147 to maintain complex I activity, thereby protecting tumor cells from mitochondrial-dependent apoptosis under metabolic stress and facilitating metabolic adaptation [45]. Conversely, LSM7 is an RNA-binding protein primarily involved in mRNA processing and alternative splicing. Recent breast cancer studies have reported that *LSM7* is highly expressed in metastatic tissues and promotes cell migration, invasion, and lung metastasis through the regulation of *CD44* alternative splicing, thereby contributing to poor prognosis [46]. Pan-cancer analyses further corroborated that *LSM7* upregulation is linked to poorer survival outcomes across multiple tumor types [47]. The co-occurrence of *NDUFS6* and *LSM7* upregulation and high *BAX* expression likely reflects coordinated adaptive processes involving mitochondrial metabolism and post-transcriptional RNA regulation in prostate tumor cells. However, because molecular studies investigating these genes in prostate cancer are limited, and our data did not demonstrate a significant association between their expression and survival outcomes, their specific clinical relevance and underlying mechanisms remain to be elucidated and warrant further investigation through targeted functional studies.

The limitations of this study should be acknowledged. First, the analyses relied largely on retrospective clinical data and publicly available transcriptomic datasets, and were conducted in a predominantly Taiwanese study population. Consequently, the generalizability of these findings to other ethnic groups may be restricted by differences in genetic background, varying allele frequencies, and unmeasured lifestyle confounders. Second, our targeted investigation of a preselected set of candidate SNPs may have limited the identification of relevant variants outside the ER stress and UPR pathways. Third, the observational nature of this study limits causal inference; mechanistic interpretations of pathway activity and gene regulation remain hypothetical without direct functional validation. Therefore, these findings should be interpreted with caution and require validation in larger, multiethnic prospective cohorts and rigorous molecular experiments.

## 5. Conclusions

Our results indicate that *BAX* rs182509214 is strongly associated with prognosis following ADT in patients with prostate cancer and that *BAX* is broadly overexpressed in prostate tumor tissues relative to normal tissues. These findings highlight the potential of *BAX* as a candidate biomarker for prognostic risk stratification and provide a foundation for future mechanistic studies exploring therapeutic targets within the ER stress pathway. Nonetheless, the clinical utility of *BAX*-based biomarkers must be definitively established through large-scale, multiethnic validation studies and direct experimental confirmation.

## Figures and Tables

**Table 1:** Association between SNPs in the ER stress pathway and overall survival in patients undergoing ADT.

Gene	SNP	Minor	Major	MAF	Frequency ^1^	HR (95% CI)	*p*	FDR
*BCL2L11*	rs616130	C	A	0.425	118/299/213	1.20 (1.05–1.37)	0.006	0.214
*BAX*	rs182509214	G	T	0.037	0/46/582	1.60 (1.13–2.27)	0.008	0.214

Abbreviations: SNP, single-nucleotide polymorphism; ER, endoplasmic reticulum; ADT, androgen deprivation therapy; MAF, minor allele frequency; HR, hazard ratio; CI, confidence interval; FDR, false discovery rate. ^1^ Number represents minor allele homozygotes/heterozygotes/major allele homozygotes.

## Data Availability

Data will be available on reasonable request.

## Supplementary Materials

The following supporting information can be downloaded at https://www.mdpi.com/article/10.3390/genes17070804/s1: Table S1: Clinical and pathological characteristics of the study population and their association with overall survival.
