# Supplementary material for: Integrative Genomic and Transcriptomic Analysis Identifies BAX as a Prognostic Marker of Disease Progression in Prostate Cancer
Source: Genes (Basel). 2026 Jul 15;17(7):804. doi: 10.3390/genes17070804 (PMC13409293; doi:10.3390/genes17070804)
Supplement: Supplementary file 1 [file genes-17-00804-s001.zip › genes-4372379-supplementary.pdf]

Table S1. Clinical and pathological characteristics of the study population and their association with overall survival

| Characteristics | Total, n (%) | Deaths, n (%) | Median, months | *p* |
|---|---|---|---|---|
| Total, n | 630 | 414 | 108.5 | |
| Age at diagnosis, years | | | | |
| Median (IQR) | 73 (67-79) | | | |
| <74 | 344 (54.7) | 201 (48.7) | 128.1 | <0.001 |
| ≥74 | 285 (45.3) | 212 (51.3) | 85.9 | |
| PSA at ADT initiation, ng/mL | | | | |
| Median (IQR) | 34.5 (11.25-129) | | | |
| <35 | 307 (50.6) | 167 (41.9) | 137.8 | <0.001 |
| ≥35 | 300 (49.4) | 232 (58.1) | 71.7 | |
| Clinical stage at diagnosis | | | | |
| T1/T2 | 187 (29.9) | 103 (25.1) | 137.6 | <0.001 |
| T3/T4/N1 | 205 (32.8) | 119 (29.0) | 137.8 | |
| M1 | 233 (37.3) | 189 (46.0) | 58.8 | |
| Gleason score at diagnosis | | | | |
| 2-6 | 188 (30.6) | 112 (27.7) | 132.7 | <0.001 |
| 7 | 195 (31.7) | 115 (28.5) | 121.1 | |
| 8-10 | 232 (37.7) | 177 (43.8) | 62.6 | |
| PSA nadir, ng/mL | | | | |
| Median (IQR) | 0.14 (0.01-1.16) | | | |
| <0.15 | 314 (50.7) | 167 (41.1) | 158.8 | <0.001 |
| ≥0.15 | 305 (49.3) | 239 (58.9) | 58.8 | |
| Time to PSA nadir, months | | | | |
| Median (IQR) | 11 (5-20) | | | |
| <12 | 323 (52.2) | 216 (53.2) | 76.6 | <0.001 |
| ≥12 | 296 (47.8) | 190 (46.8) | 122.9 | |

Abbreviations: IQR, interquartile range; PSA, prostate-specific antigen; ADT, androgen deprivation therapy.

Totals may not sum to the overall number of patients because of missing data.
